# Progressive Wound Necrosis Associated With Postoperative Thrombocytosis in Mastectomy and Immediate Breast Reconstruction Surgery: Report of a Case

**Published:** 2009-08-20

**Authors:** Robert X. Murphy, Ginger A. Holko, Afifi A. Khoury, Aaron D. Bleznak

**Affiliations:** Lehigh Valley Health Network, Allentown, Pennsylvania

## Abstract

A 37-year-old who underwent splenectomy for motor vehicle accident-related injuries was diagnosed with stage IIA carcinoma of left breast 12 years later. She underwent bilateral mastectomy and bilateral immediate unipedicle TRAM flap reconstruction. Her preoperative platelet counts ranged from 332 to 424 K/cmm. Intraoperative fluorescein confirmed mastectomy flap viability. On postoperative day 1, platelet count was 374 K/cmm and all suture lines appeared benign. The patient was discharged 3 days later with healthy appearing tram flaps and slight epidermolysis in the abdominal region. Over the next 2 weeks, both the mastectomy flaps and the abdominal region underwent progressive necrosis as the platelet count increased to 1390 K/cmm. Aspirin therapy was instituted at this time. The TRAM flaps remained completely viable. Eighteen days later, the patient required wound debridement with secondary closure of the breast wounds. Platelet count peaked at 1689 K/cmm 2 days later (postoperative day 38). The wounds deteriorated again and were managed conservatively. Two months after mastectomy, the first area of spontaneous healing was documented (platelet count 758 K/cmm). Ultimately, wounds healed as platelet count reached its preoperative baseline. We hypothesize that an abnormal secondary thrombocytosis at subdermal plexus level caused problematic healing in this patient's mastectomy and abdominal flaps.

Thrombocytosis, a condition in which there is an abnormally high number of circulating platelets, can be described as either primary or secondary. Primary thrombocytosis is usually associated with myeloproliferative disorders such as polycythemia vera and essential thrombocythemia.^1^,^2^ Secondary thrombocytosis occurs after trauma, infection, splenectomy, or as a reaction to drugs.^3^–^5^ In these situations, the platelet count routinely increases at least 30%, peaking at 1 to 3 weeks and returning to normal over a period of 2 weeks to several months.^6^

Traditionally, the risk of post-splenectomy thromboembolic phenomenon has been considered quite low with few reports of complications appearing in the literature. Herein, we report an unusual case of a young woman who underwent bilateral mastectomy with immediate unipedicle TRAM flap reconstruction and experienced progressive wound complications associated with significant platelet count increases.

## CASE REPORT

A 25-year-old Caucasian female underwent a splenectomy which was performed through a vertical midline incision in 1994 for injuries sustained in a motor vehicle collision. Twelve years later, at age 37, she was diagnosed with stage IIA carcinoma of the left breast. She presented for bilateral mastectomies with immediate autologous tissue reconstruction. She had completed four cycles of neoadjuvant dose-dense adriamycin and cytoxan chemotherapy 2 months previously. Following her chemotherapy, her platelet counts ranged from 332 to 424 K/cmm. Her medical history was significant for obesity (BMI 40.4) and asthma. The patient had smoked 1 pack per day for 15 years, but had discontinued that practice during her chemotherapy treatment.

**Figure 1:**
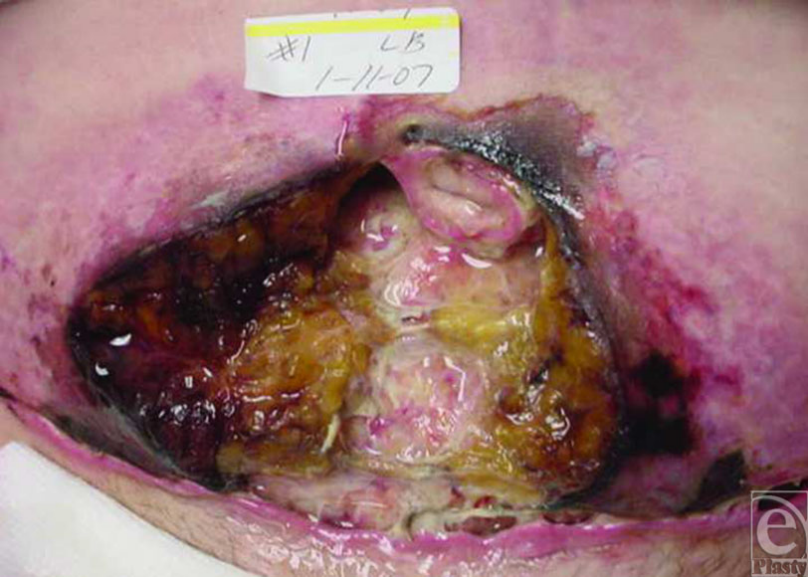
Preoperative view of necrotic abdominal wound.

**Figure 2:**
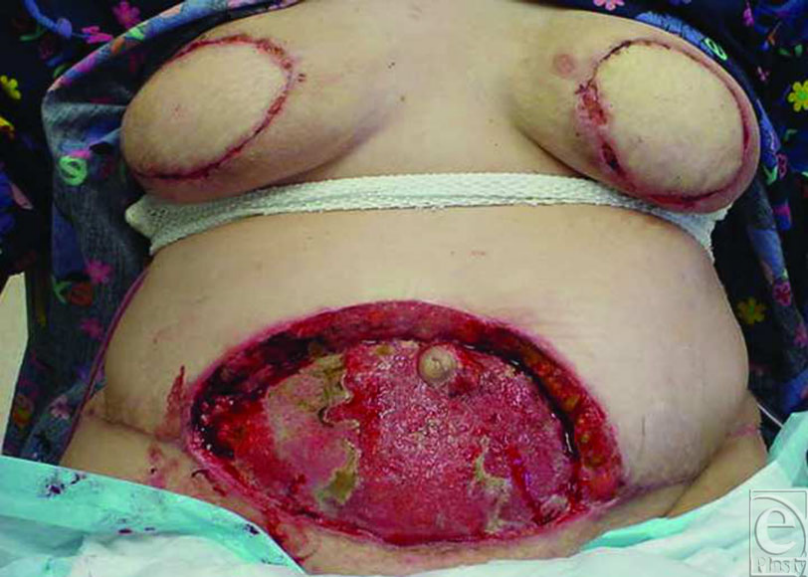
Postoperative day 8 view of deteriorating wounds after debridement of abdominal and chest wounds and closure of breast wounds.

**Figure 3:**
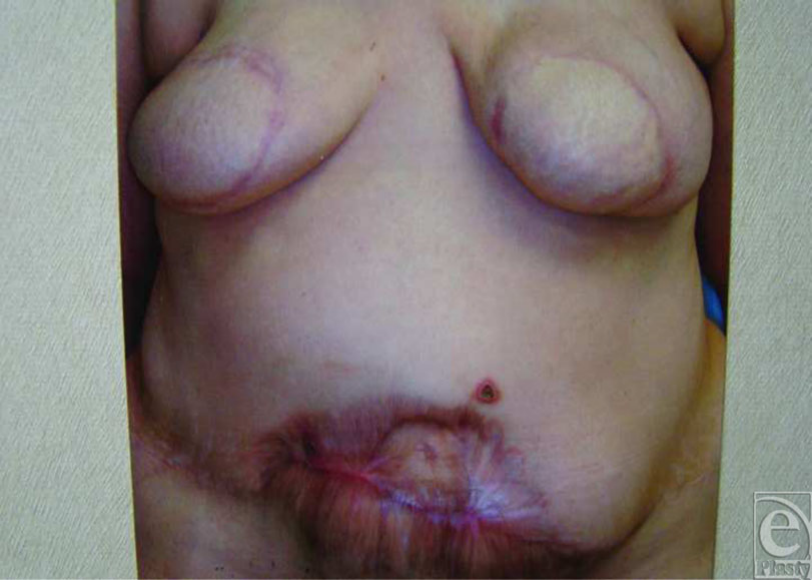
View of wounds after healing by secondary intention.

In 2006, she underwent a bilateral mastectomy with immediate bilateral ipsilateral single pedicle transverse rectus abdominis myocutaneous (TRAM) flap reconstruction. Venous thromboembolism prophylaxis was provided intraoperatively and during her hospital stay via sequential compression devices (SCD). Although not a routine component of our customary reconstructions, fluorescein was utilized intraoperatively to confirm the viability of the mastectomy flaps prior to final insetting of the TRAM flaps. The patient did well during the procedure and suffered no intraoperative or postoperative hypotensive episodes. On postoperative day 1, her wounds looked well and her platelet count was 374 K/cmm. She was discharged on postoperative day 4 with slight epidermolysis in the abdominal flap just cephalad to the mid-line sutures. Over the next 2 weeks, both the mastectomy and abdominal skin flaps underwent progressive necrosis as the platelet count rose to 1390 K/cmm. The patient's hematologist/oncologist was consulted at this point. The consultant's opinion was that this was a reactive phenomenon and required no additional testing or intervention. Even though the TRAM flaps remained completely viable, aspirin therapy was instituted empirically. The necrosis of the mastectomy and abdominal flaps continued to evolve and on postoperative day 18, the patient underwent operative debridement of her abdominal (Fig 1) and chest wounds. The breast wounds were closed primarily and negative pressure wound therapy (NPWT) was applied to her abdomen. Intraoperative findings confirmed extensive skin subcutaneous necrosis in the mastectomy and abdominal flaps, whereas the TRAM flaps had minimal evidence of peripheral fat necrosis and no areas of skin loss. Her platelet count peaked at 1689 K/cmm 2 days later. Over the next several weeks, the wounds continued to deteriorate, but the patient elected to have them managed conservatively (Fig 2). The patient was followed at weekly intervals in the outpatient clinic. Two months after her original surgery the platelet count had decreased to 758 K/cmm, and the first area of spontaneous healing in the form of healthy granulation and wound contraction was documented. Ultimately, the wounds healed by secondary intention (Fig 3) as the platelet count returned to its postoperative nadir of 530.

## DISCUSSION

Thrombocytosis is considered to exist when the concentration of circulating platelets reaches 400 to 600 K/cmm.^7^,^8^ Although it has been suggested that platelet counts in excess of 1 million/cm are necessary for hemorrhage and thrombotic sequelae,^9^ there is no consensus on scientifically derived, evidence-based indications for antiplatelet therapy in the perioperative period. In part, this may be due to a lack of correlation between platelet counts and thrombocytosis.^8^,^10^–^14^

The surgical literature has implicated thrombocytosis as a potential etiologic factor in phlebitis and deep venous thrombosis (DVT),^15^ pulmonary embolism,^16^ mesenteric ischemia,^17^,^18^ and cerebrovascular accidents^9^ and infarction.^19^ However, Kuo and colleagues found no evidence to suggest that thrombocytosis in and of itself had any negative impact on microvascular patency rates in a splenectomy-induced thrombocytosis model. Traditionally, therefore, presence of thrombocytosis had not warranted routine anticoagulation prophylaxis.^8^

Recent studies have investigated the biochemistry of the platelet and its response in the perioperative period. As early as the 1950s and 1960s, it was recognized that platelet levels increased after surgery.^20^–^22^ Folman et al.^23^ identified thrombopoietin and other cytokines as stimulators of postoperative thrombopoiesis. Yet it was not until the mid 1970s that the functional status of the platelet was found to be an even more important factor than the absolute number. Matsuda et al.^24^ reported two cases of postoperative thrombocytosis in which the platelet populations were studied. By virtue of their in vitro assays, they were able to identify two populations of platelets in the thrombocytotic postoperative situation. Each population was associated with a distinct phase of aggregation, the secondary population whose functions were much more enhanced than the first. It was hypothesized that it is this secondary population that is responsible for the postoperative thromboembolic phenomenon. Interestingly, the enhanced functioning of this platelet group was easily suppressed by the low-dose aspirin.

In light of the findings of a large multicenter trial that determined that aspirin did, in fact, reduce the risk of pulmonary embolism and DVT after surgery,^25^ Matsuda et al.^24^ recommend that aspirin be employed to prevent significant thromboembolic complications, particularly when platelets are excessively produced and hyper-functioning. Twenty-five years later, Kuo et al.^7^ echoed this sentiment by concluding that “a platelet activation profile study is even more important than platelet count alone in preoperative evaluation of microvascular surgery cases. Once both thrombocytosis and platelet activation occur, antiplatelet activation drugs are recommended.”

This patient had an uncomplicated mastectomy procedure and underwent intraoperative fluorescein testing to ensure the viability of all skin flaps. Despite this maneuver, all of the native skin flaps necrosed, while the musculocutaneous flaps survived. Although epidermolysis was noted early in the postoperative period, extensive necrosis became evident as the platelet count exceeded 1000 K/cmm as suggested by Heller et al.^9^ Even though aspirin therapy was instituted, the extensive nature of tissue necrosis required that the patient be returned to the operating room for debridement. At the second procedure, all wounds were debrided back to healthy, vascularized tissue. Despite this, all three wounds necrosed as the platelet count peaked at 1689 K/cmm. UItimately, as the platelet count resolved, the wounds responded to NPWT and healed by secondary intention. Since the TRAM flaps survived, we hypothesize that operative trauma to the small vessels in the skin flap subdermal plexus caused an aggregation of an abnormally high number of platelets, a secondary thrombocytosis, resulting in small vessel occlusion and subsequent skin necrosis. Should this patient, or similar patients, require surgery in the future, a platelet activation profile study might be a valuable tool to assess the potential need for aggressive anti-platelet therapy.

## CONCLUSION

The series of events in this case may have resulted from abnormally robust platelet aggregation (secondary thrombocytosis) at the subdermal plexus level in the most vulnerable distal areas of the flaps. Adequate hematologic evaluation may be prudent in the face of significant preoperative thrombocytosis.
